# Neural regions discriminating contextual information as conveyed through the learned preferences of others

**DOI:** 10.3389/fnhum.2015.00492

**Published:** 2015-09-08

**Authors:** Su Mei Lee, Gregory McCarthy

**Affiliations:** Department of Psychology, Yale UniversityNew Haven, CT, USA

**Keywords:** context discrimination, expectations, pSTS, fMRI, MVPA

## Abstract

The human brain consists of a network of regions that are engaged when one observes the movements of others. Observing unexpected movements, as defined by the context, often elicits greater activity, particularly in the right posterior superior temporal sulcus (pSTS). This implies that observers use contextual information to form expectations about an agent’s goal and subsequent movements. The current study sought to identify regions that support the formation of these context-dependent expectations, with the pSTS being one candidate, given the consistent contextual modulation of its activity. We presented participants with fictitious individuals who had emotion-dependent food preferences, and instructed participants to indicate which food they expected each individual to choose based on the individual’s current emotional state. Each individual’s preference and emotional state therefore created a context that informed the observer’s expectation of the individual’s choice. Multi-voxel pattern analysis (MVPA) was used to assess if these different contexts could be discriminated in the pSTS and elsewhere in the brain. No evidence for context discrimination was found in the pSTS. Context discrimination was found instead a network of other brain regions including the anterior medial prefrontal cortex (amPFC), bilateral parietal cortex, left middle temporal gyrus (L MTG) and left anterior temporal lobe (L ATL), which have been previously associated with context processing, and semantic and memory retrieval. All together, these regions possibly support the formation of context-dependent expectations of an agent’s goal.

## Introduction

The human brain consists of a network of regions that are engaged when one observes the movements and actions of other living things. These regions are involved in processing the form and kinematics of motion, and identifying the actions performed (Thompson and Parasuraman, 2012). The brain, however, does not merely react to observed movements, but also seems to predict the movements of an agent, based on inferred goals and intentions. Evidence for this idea comes from studies showing that the same observed movements elicit greater activity, particularly in the right posterior superior temporal sulcus (pSTS), when the context renders the movement unexpected than expected. For example, Pelphrey et al. (2003) found that pSTS activity to shifts in an avatar’s eye gaze was greater when the gaze shift did not occur in the direction of a preceding flashing checkerboard than when it did. In another study, Brass et al. (2007) found greater pSTS activity to the same action when the action seemed implausible than when it seemed plausible, for example, an actress flipping a light switch with her knees when her hands were free compared to when her hands were occupied. Vander Wyk et al. (2009, 2012) found greater pSTS activity when an actress’ action was incongruent with her expressed emotion (i.e., reaching *toward* a cup that she had previously expressed dislike for) than when it was congruent (i.e., reaching *away* from the cup that she had previously expressed dislike for). Increased pSTS activity to actions that are unexpected has been found in other studies as well (Pelphrey et al., 2004; Saxe et al., 2004; Shultz et al., 2011).

The differences in neural response to observing identical actions embedded within different contexts suggests several stages of processing. That the pSTS shows different responses to expected and unexpected actions necessitates that the observer must have first formed an expectation about the agent’s goal. Forming an accurate expectation, in turn, depends on the observer having assessed the context preceding the action. Indeed, according to the predictive coding framework of action observation, context provides priors from which predictions about an agent’s intentions are formed, which in turn informs predictions about the immediate goal of an agent’s subsequent movements, and the kinematics of those movements (Kilner et al., 2007). Therefore, it seems that assessing context and forming expectations about intentions and goals can occur *prior* to observing an action. Here, our operational definition of context is any situation-specific information that informs an observer’s expectation of an agent’s intention. For example, in Vander Wyk et al. (2009), the actress’ particular emotional expression directed at a particular cup served as the contextual information that allowed the observer to expect that she would either choose that cup or the other cup. What are the neural substrates of these earlier stages of processing? That is, which regions are involved in assessing the context, thus allowing the observer to predict an agent’s goal?

To investigate this question, we reasoned that if a brain region uses contextual information to inform expectations about an agent’s goal, then this region should be able to discriminate between different contexts. Therefore, in this study, participants were presented with unique contexts that led to specific expectations. To avoid using spatial cues as context, as the pSTS has also been implicated in attention reorienting (Corbetta et al., 2008), participant’s expectations were instead informed via learned preferences of fictitious individuals. To this end, we used an ecologically valid manipulation of assigning different food preferences to these fictitious individuals depending on their emotional state (Lyman, 1982). Specifically, one individual would choose to eat meat when he was happy, and vegetables when he was sad. The other individual had the opposite preference. During the experimental task, participants were presented with each individual and his current emotional state, and were asked to indicate which food they expected the individual to choose based on the individual’s current emotional state. Each individual’s preference and his current emotional state therefore created a context that would inform the observer’s expectation of the individual’s choice. Multi-voxel pattern analysis (MVPA) was used to assess if these different contexts could be discriminated from one another. Unlike previous studies, neither spatial cuing in the form of motion nor outcome was presented in this study because our aim was to investigate context assessment and expectation formation prior to observing an outcome.

Given the robust and consistent influence of context on pSTS activity reported in the literature, the pSTS served as a region-of-interest (ROI) on which we performed a targeted analysis. The role of assessing context is also plausible for this region given that the surrounding cortex in the inferior parietal lobules has been proposed as a convergence zone for multimodal contextual information to support semantic (Binder and Desai, 2011) and episodic (Shimamura, 2011) memory. However, it is also possible that contextual information is represented not in the pSTS, but in other regions. In particular, the medial prefrontal cortex (mPFC) has been suggested to use contextual associations to form predictions about possible subsequent stimuli (Bar, 2009). We therefore also conducted a whole-brain searchlight analysis to uncover other brain regions that could discriminate between contexts.

## Materials and Methods

### Participants

Twenty-one right-handed, healthy adults (14 male, mean age 23.2 ± 3.9 years) participated in the study. All participants had normal or corrected-to-normal vision and had no history of neurological or psychiatric illnesses. The protocol was approved by the Yale Human Investigation Committee and all participants gave informed consent. Data from one participant was excluded because the timing files were corrupted, and from another participant because of excessive artifacts in the data. Therefore, results from nineteen participants are reported.

### Stimuli and Design

Stimuli consisted of colored pictures of three male faces with neutral expressions, obtained from the NimStim database (Tottenham et al., 2009), along with 36 colored pictures of meat dishes and 36 colored pictures of vegetable dishes obtained from the Internet. Stimuli were presented with using Psychtoolbox 3.0.8 (Brainard, 1997; Pelli, 1997) in MATLAB 7.8 (The MathWorks, Inc., Natick, MA, USA).

**Figure 1:**
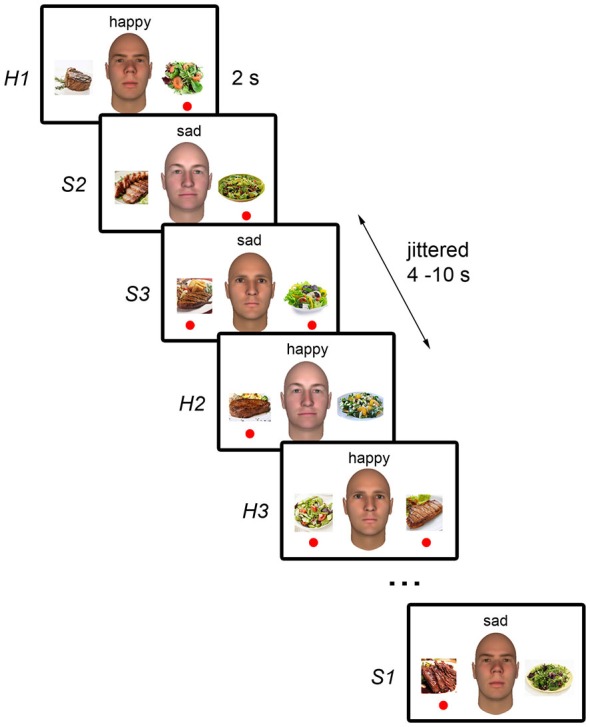
**Schematic illustration of the experimental paradigm**. During each trial, the neutral face picture of one of the three white male individuals was displayed. As the NimStim faces used cannot be published, sample faces generated with FaceGen (Singular Inversions, Toronto, ON, Canada) are shown here instead. Pictures of a meat dish and a vegetable dish were presented on the left and right of the face. The word “happy” or “sad” was displayed above the face to indicate the individual’s current emotional state. Participant’s task was to indicate, using the left and right button presses, which dish the individual would choose based on the individual’s emotional state. Trials were presented for 2 s and were separated by a 4–10 s jittered interval during which a fixation cross was displayed (not shown). The red circles indicate each individual’s emotion-dependent food preferences and were not displayed during the task.

The stimuli were presented using an event-related design. In each trial, one of the three faces was presented along with a text cue above the face indicating the person’s emotional state (“happy” or “sad”), and pictures of a meat dish and a vegetable dish on the left and right of the face (Figure 1). Each trial was presented for 2 s and trials were separated by a 4–10 s jittered fixation interval. Each run consisted of six trials per condition (i.e., each face paired with each emotion) to give a total of 36 trials per run, and a run duration of 5 min. The program “optseq2”^1^ was used to generate the optimal sequence and separation of trials for maximal statistical efficiency of rapid-presentation event-related hemodynamic response estimation for each run (Dale, 1999). The position of the meat and vegetable dishes on the left and right of the face was counterbalanced across trials within each condition and each run. Ten runs were presented.

### Experimental Procedure

Prior to scanning, participants were introduced to three fictitious male individuals (“John”, “Alex”, and “Rick”). They were briefed that each individual had different food preferences depending on their emotional state. When John was happy (“H1”), he would choose to eat vegetables, but when he was sad (“S1”), he would choose to eat meat. The exact description presented to participants read, “This is John. He is into healthy living so when he’s feeling happy, he’ll choose to eat vegetables because they are refreshing. However, when he’s sad, he’ll indulge and choose to eat meat instead.”

Alex, however, had the opposite preference; he would choose to eat meat when he was happy (“H2”), but vegetables when he was sad (“S2”). The description of Alex read, “This is Alex. Unlike John, when he’s happy, he’ll indulge and choose to eat hearty meat meals. However, when he’s sad, he’ll want something refreshing so he’ll choose vegetables instead.” These two individuals had opposite preferences so that the discrimination of context would not be confounded with discrimination of emotion (i.e., happy vs. sad) or food choice (i.e., meat vs. vegetables). These trials were considered the “Preference” trials, where participants had to rely on information about each person’s preference and emotional state to form expectations about their choice.

Rick had no particular preference and could choose to eat either meat or vegetables when he was happy (“H3”) or sad (“S3”). The description of Rick read, “This is Rick. He doesn’t have a strong preference for either type of food. When he’s happy, he some times chooses to eat meat and he some times chooses to eat vegetables. Likewise, when he’s sad, he some times chooses to eat meat and he some times chooses to eat vegetables.” These were the “No Preference” trials and served as control trials since there was no contextual information from which the participants could form an expectation about the person’s choice.

Participant’s task in the scanner was to indicate on each trial, using their right index and middle fingers corresponding to the left and right response buttons respectively, which food item they expected each person would choose based on his emotional state. No feedback was given during the in-scanner task. However, participants were familiarized with the preferences by performing a practice task, which included feedback, until they achieved an accuracy of at least 75%.

A 2 (Person: John, Alex) × 2 (Emotion: Happy, Sad) repeated measures analysis of variance (ANOVA) revealed that participants performed equally well in the “Preference” trials for which there were correct answers (*M* = 93.7%); there was no main effect of Person or Emotion, and no Person x Emotion interaction (all *p*s > 0.5). However, there was a marginal main effect of Emotion on response times (*F*_(1,72)_ = 3.078, *p* = 0.084); participants took longer to respond to Sad trials (*M* = 1486 ms) than to Happy trials (*M* = 1362 ms).

### Image Acquisition and Preprocessing

Data were acquired using a 3T Siemens TIM Trio scanner with a 32-channel head coil. Functional images were acquired using a multi-band echo-planar pulse sequence (TR = 1000 ms, TE = 30 ms, flip angle = 62°, FOV = 210 mm, matrix = 84 × 84, slice thickness = 2.5 mm, 51 slices). Two structural images were acquired for registration: T1 coplanar images were acquired using a T1 Flash sequence (TR = 285 ms, TE = 2.61 ms, flip angle = 70°, FOV = 220 mm, matrix = 192 × 192, slice thickness = 2.5 mm, 51 slices), and high-resolution images were acquired using a 3D MP-RAGE sequence (TR = 2530 ms, TE = 3.31 ms, flip angle = 7°, FOV = 256 mm, matrix = 256 × 256, slice thickness = 1 mm, 176 slices).

Image preprocessing was performed using the FMRIB Software Library (FSL).^2^ Structural and functional images were skull-stripped using the Brain Extraction Tool (BET). The first six volumes (6 s) of each functional dataset were discarded to allow for MR equilibration. Functional images then underwent motion correction (using the MCFLIRT linear realignment) and high-pass filtering with a 0.01 Hz cut-off to remove low-frequency drift. No spatial smoothing was applied to the functional data. The functional images were registered to the coplanar images, which were in turn registered to the high-resolution structural images for subject-level analyses. Subject-level results were later normalized to the Montreal Neurological Institute’s MNI152 template, using non-linear registration, for group-level analyses.

### Multi-Voxel Pattern Analysis (MVPA)

To obtain data samples for the classification analysis, participant’s preprocessed functional data were first normalized to their structural image (which were resampled to the resolution of the functional data) using the transformation matrix from preprocessing. Regression analyses were then performed to obtain beta estimates for each trial, using least-squares-sum estimation (AFNI’s 3dLSS), which is recommended for classification analyses involving fast event-related designs (Mumford et al., 2012). The model consisted of separate regressors for each 2-s trial from each condition, convolved with a hemodynamic response function, along with the six motion parameters obtained from preprocessing as nuisance regressors. Estimates were obtained for each run separately, and then concatenated to form a beta series for each participant. All classification analyses were implemented using PyMVPA (Hanke et al., 2009) using a Gaussian Naïve Bayes (GNB) classifier and a leave-one-run-out cross-validation scheme. Only correct trials were included in the analysis and PyMVPA’s Balancer function was used to ensure an equal number of trials across conditions for each cross-validation fold. To determine if a region could discriminate between the different contexts, we used a GNB classifier to perform a four-way classification to discriminate correct “Preference” trials (i.e., H1, S1, H2, S2).

A significant four-way classification can arise from accurate classification of some categories but not others. Therefore, we focused our discussion on regions where the classifier made the correct prediction about the actual target category on majority of the trials, that is, where the diagonal elements of the confusion matrix had the highest numerical value in each row. For each participant, the confusion matrices from all voxels within each searchlight cluster were averaged. The mean confusion matrix was then scaled such that each cell in the resulting confusion matrix reflected the percentage of trials in each category that were classified as each of the four potential categories (e.g., percentage of H1 trials classified as H1 trials, S1 trials, H2 trials, S2 trials). The cells in each row therefore add up to 100 (or approximately 100 due to rounding). The group-level confusion matrix for each searchlight cluster was obtained by averaging the subject-level confusion matrices.

To verify that a successful four-way classification of the “Preference” trials indeed reflected context-dependent expectations (i.e., each individual’s preferences and their emotional state), we also conducted a two-way classification on the control “No Preference” trials (i.e., H3, S3). Here, we expected that these trials should not be successfully discriminated since there was no preference and therefore no contextual information from which participants could form an expectation about the individual’s choice. Only trials with behavioral responses were included in the analysis (i.e., missed trials were excluded).

#### ROI-Based MVPA

An independent pSTS ROI was obtained from the Atlas of Social Agent Perception (Engell and McCarthy, 2013). Briefly, this Atlas included results from a Biological Motion localizer (consisting of blocks of point-light figures and blocks of their scrambled counterparts) that was run on 121 participants. The probability map of the Biological Motion > Scrambled Motion contrast, which localizes the pSTS, was thresholded at 0.1 and intersected with the right Supramarginal Gyrus from the Harvard Oxford Atlas to obtain a liberal pSTS mask. The mask was further edited manually to remove voxels spreading into the parietal operculum. The resulting ROI of 751 voxels (Figure 1, in yellow) was then transformed into subject-space for each participant.

The beta estimates within the ROI were mean-normalized by *z*-scoring within each sample to remove mean differences between samples. Feature selection was performed on the samples in the training set of each cross-validation fold by conducting a one-way ANOVA on the beta estimates for the four “Preference” trials for each voxel in the pSTS ROI. The top 123 voxels (to match the number of voxels used for the searchlight analysis described later) that showed the greatest variance between the four trial types were selected as features for that cross-validation fold. The accuracies from all participants were then averaged to obtain the group level classification accuracy.

Significance testing at the group level was implemented using a combination of permutation and bootstrap sampling methods (Stelzer et al., 2013). Specifically, the data labels for each participant were permuted (within each run) 100 times and the classification analysis was repeated using each permuted label set to yield 100 chance accuracies for each participant. We then randomly drew one of the chance accuracies from each participant and averaged these accuracies to obtain a chance group-level accuracy. This random sampling (with replacement) was repeated 10^5^ times to create a group-level null distribution. The true group-level classification accuracy was then compared to the null distribution to obtain the *p*-value associated with the accuracy.

#### Whole-Brain Searchlight Analysis

To identify other brain regions that discriminate context-specific information, we conducted a whole-brain searchlight analysis in subject-space for each participant with a three-voxel-radius searchlight consisting of 123 voxels centered on every non-zero voxel in an MNI152 brain mask. The four-way classification analysis performed for each searchlight followed the method used in the ROI-based analysis, except that no feature selection was conducted. The classification accuracy for each searchlight was assigned to the voxel at the center of the searchlight, yielding a whole-brain classification accuracy map for each participant. Each participant’s accuracy map was transformed back into MNI152 template space. The group-level classification accuracy map was obtained by averaging the accuracy maps from all participants.

Significance testing of the whole-brain classification results also used permutation and bootstrap sampling methods, along with cluster thresholding to correct for multiple comparisons (Stelzer et al., 2013). Specifically, we ran the searchlight classification analysis for each participant an additional 100 times, each time using a random permutation of the data labels (within each run), thus producing an accuracy map of chance classification. Each participant therefore had 100 chance accuracy maps. Each of these maps was then normalized to the MNI152 template space. To obtain a null distribution for the group level classification accuracies, we generated 10^5^ group-level chance accuracy maps, each of which was obtained by choosing a random chance accuracy map from each participant and averaging those randomly chosen maps. A whole-brain threshold of *p* < 0.001 at each voxel was then applied to the group-level accuracy map.

Cluster thresholding was used to correct for multiple comparisons. Each of the 10^5^ group-level chance maps were also thresholded at voxel-wise *p* < 0.001. We recorded the number of clusters for each cluster size occurring in each of these 10^5^ thresholded chance maps and generated a null distribution of clusters. Each recorded cluster across all 10^5^ chance maps was then assigned a *p*-value based on the occurrence of its size in the chance-level cluster distribution. Significant clusters were those whose probability survived a false discovery rate (FDR) of *q* < 0.05. To verify that the significant four-way classification reflected accurate discrimination of all four categories, a cluster-level confusion matrix was obtained by averaging the confusion matrices of all searchlights in each significant cluster.

We also conducted a whole-brain searchlight analysis performing a two-way classification using the two “No Preference” trials in each searchlight to verify that regions that discriminated the four “Preference” trials did not also discriminate the two “No Preference” trials.

## Results

### Classification Analysis on the pSTS Region-of-Interest (ROI)

No significant four-way classification of “Preference” trials was found in the pSTS ROI (*M* = 25.49%, *p* = 0.314). There was also no significant two-way classification for the control “No Preference” trials (*M* = 48.89%, *p* = 0.792). To assess if the four-way classification would improve with a larger number of features, the classification analysis was also run with the top 200, 300, and 400 voxels from the feature selection, but no improvement in the four-way classification accuracy was found (200 voxels: *M* = 25.36%, 300 voxels: *M* = 25.34%, 400 voxels: *M* = 25.71%, all *p*s > 0.2). We also performed a separate two-way classification, using only “Preference” trials, to assess if the pSTS could discriminate the expected outcome (in this case food choice, i.e., meat vs. vegetables). No successful discrimination of expected outcome was found with any feature selection size (all *p*s > 0.5).

### Whole-Brain Searchlight Analysis

**Figure 2:**
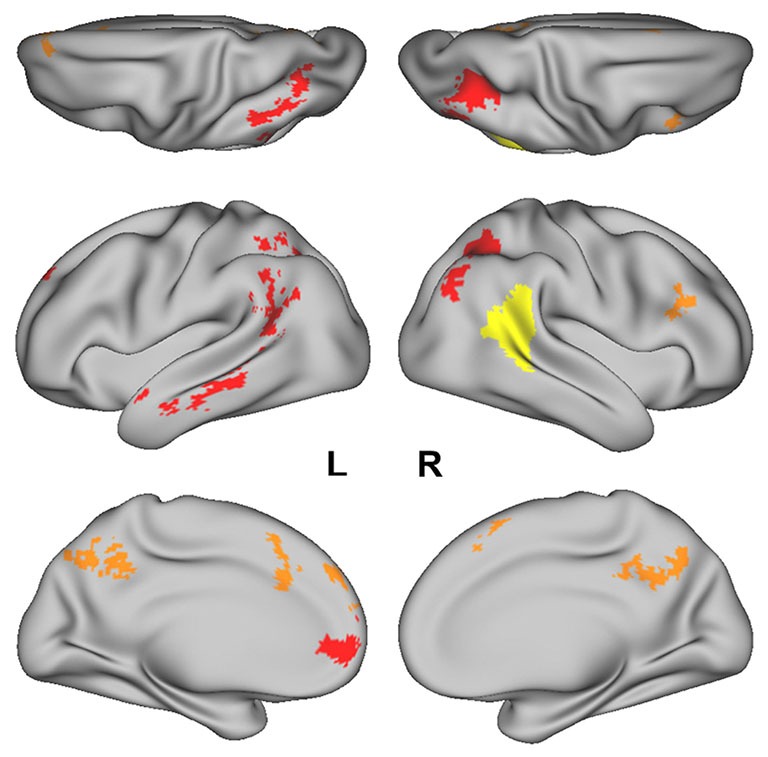
The posterior superior temporal sulcus (pSTS) region-of- interest (ROI) obtained from the Atlas of Social Perception (Engell and McCarthy, 2013) for the ROI-based MVPA is displayed in yellow. Clusters of searchlight centers with significant four-way classification of the “Preference” trials in the whole-brain searchlight analysis are displayed in red and orange. Regions in red (i.e., L IPL/IPS, R IPS, amPFC, L MTG, and L ATL) had confusion matrices in which the diagonal elements had the highest numerical value in each row.

**Figure 3:**
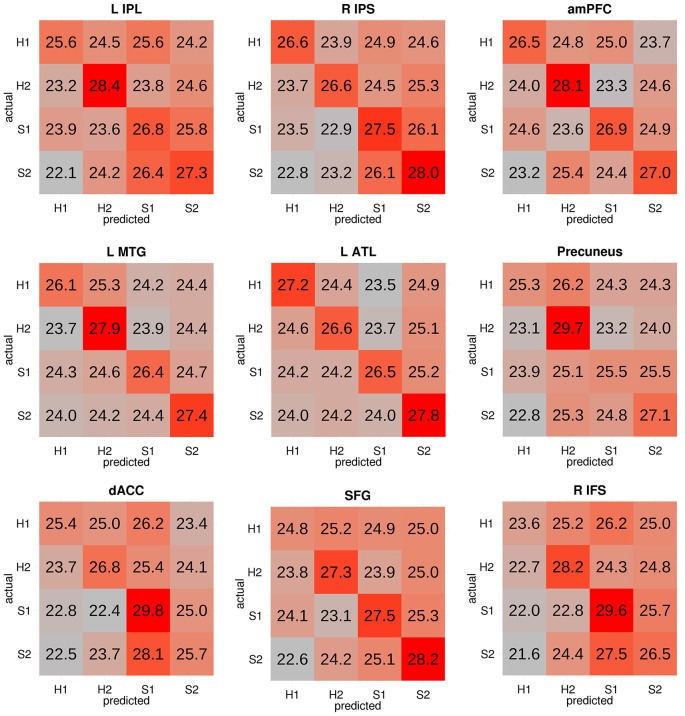
**Confusion matrices from each significant cluster from the four-way classification**. Each cell reflects the group-level proportion of each type of trial (in rows) that were classified as each of the four types of trials (in columns). The cells in each row therefore add up to 100 (or approximately 100 due to rounding). Cells are colored according to a gradient ranging from the lowest (gray) to highest numbers (red). Successful classification of all four categories is reflected through strong red colors in the diagonal from top left to bottom right. The first five regions (i.e., L IPL, R IPS, amPFC, L MTG, and L ATL) had confusion matrices in which the diagonal elements had the highest numerical value in each row. H1: John-happy, H2: Alex-happy, S1: John-sad, S2: Alex-sad.

Regions that successfully discriminated the “Preference” trials in the whole-brain searchlight four-way classification analysis included the left inferior parietal lobule/intraparietal sulcus (L IPL/IPS) spanning from the angular gyrus to the intraparietal sulcus, precuneus, right intraparietal sulcus (R IPS), anterior medial prefrontal cortex (amPFC), left middle temporal gyrus (L MTG), dorsal anterior cingulate cortex (dACC), superior frontal gyrus (SFG), left anterior temporal lobe (L ATL) at the anterior MTG, and right inferior frontal sulcus (R IFS; Figure 2, in red and orange; coordinates of peaks are reported in Table 1). Of these regions, the L IPL/IPS, R IPS, amPFC, L MTG, and L ATL (Figure 2, in red) yielded confusion matrices where the diagonal elements had the highest numerical value in each row (Figure 3). No regions successfully discriminated the “No Preference” trials in the whole-brain searchlight two-way classification analysis.

**Table 1:** **Coordinates of peak accuracy in each searchlight cluster**.

Region	MNI coordinates (mm)			Number of searchlights
	*x*	*y*	*z*
Left inferior parietal lobule (L IPL/IPS)*	−40	−50.5	48	553
Precuneus	5	−63	38	429
Right intraparietal sulcus (R IPS)*	32.5	−58	43	341
Anterior medial prefrontal cortex (amPFC)*	−12.5	54.5	0.5	217
Left middle temporal gyrus (L MTG)*	−62.5	−25.5	−24.5	216
Dorsal anterior cingulate cortex (dACC)	−10	19.5	35.5	163
Superior frontal gyrus (SFG)	−7.5	49.5	38	106
Left anterior temporal lobe (L ATL)*	−60	−5.5	−19.5	99
Right inferior frontal sulcus (R IFS)	47.5	19.5	20.5	82

*Regions with confusion matrices in which the diagonal elements had the highest numerical value in each row.

## Discussion

The current study sought to investigate the neural substrates of assessing contextual information to form expectations about an agent’s goal. To this end, participants were presented with fictitious individuals who had emotion-dependent food preferences, and were asked to indicate which food they expected each individual to choose given the individual’s emotional state. Here, knowledge about each individual’s emotion-dependent food preferences and the individual’s current emotional state served as a unique context that informed the observer’s expectation of the individual’s food choice (i.e., his goal). We assessed if the different contexts could be discriminated based on the spatial pattern of activity in different brain areas. Given the consistently observed influence of context of pSTS activity, the pSTS served as a ROI on which we performed a targeted analysis. We also conducted a whole-brain searchlight analysis to identify other regions in the brain that might discriminate between contexts. Despite using a liberal mask and selecting voxels that varied the most between trials to optimize classification performance, no evidence for context discrimination was found in the pSTS. However, we found robust evidence for context discrimination in three-voxel-radius searchlights centered in a network of other regions in the brain, including the left IPL/IPS, right IPS, amPFC, left MTG, and left ATL.

The positive finding in the whole-brain analysis demonstrates that our task was sensitive to our experimental manipulation, but the lack of a positive finding in the pSTS does not rule out the possibility that the pSTS may still represent contextual information. A recent study found that MVPA failed to find information about face identity in macaques, even when single-unit recordings revealed the presence of this information in the underlying neural populations (Dubois et al., 2015), demonstrating the limitations of the method. The different contexts presented in this study may not be represented in a spatially organized or consistent way in the pSTS, which is what a successful classification analysis using MVPA requires. Alternatively, the pSTS could represent contextual information, but only those conveyed through visual or other sensory modalities, as was used in previous studies, and not those conveyed through linguistic, conceptual means, as was used in this study. Similarly, the pSTS may not represent information about an agent’s stable preferences, which is only one type of contextual information, but may represent other types of contextual information that are conveyed through the stimulus, such as facial expressions. Indeed, the analysis rested on the assumption that regardless of the nature of the context, there should be a point of convergence where the contextual information is interpreted and translated into an expected outcome.

Relatedly, the searchlights that discriminated the different contexts were centered in regions associated with semantic processing and retrieval. The left ATL is involved in semantic processing (Visser et al., 2010), and has been shown to be particularly important for processing person-specific semantic information (Brambati et al., 2010), which could refer to each individual’s context-specific preferences in this study. Meta-analyses have also found that the parietal lobules and MTG regions are involved in episodic (Spaniol et al., 2009) and semantic (Binder et al., 2009) retrieval. However, previous studies that have used scenes to convey context have instead implicated the retrosplenial cortex and parahippocampal gyrus, which are associated with scene processing (Bar, 2009). The differences in regions implicated suggest that the regions that successfully discriminated the different contexts in this study may not necessarily be involved in all types of context processing, but could reflect the specific type of contextual information that is used in this task. In our study, the regions that showed successful context discrimination have previously been implicated in semantic processing and retrieval, which may reflect the retrieval of learned person knowledge required for the task. Similarly, Zaki et al. (2010) also found greater engagement of amPFC, left temporal and parietal regions when participants used contextual cues (e.g., text describing affective events) to infer a person’s emotional state than when watching a silent video of the person describing the events.

Notably, the region that was commonly implicated in both types of context studies was the amPFC, which may suggest that this region is critical for context processing more generally, regardless of domain. Indeed, the mPFC has been proposed to use contextual associations to form predictions (Bar, 2009). The mPFC has also been implicated in integrating context and past experience, albeit for guiding an organism’s responses (Euston et al., 2012). One question that can be raised from this observation is whether the same neural mechanisms are also used to guide predictions about another’s response. Interestingly, in a similar study, participants assessed how four individuals, each with different personalities, would react in a given situation (Hassabis et al., 2014). Successful discrimination of the four personalities was found in the mPFC. In our study, we also found successful within-personality discrimination, that is, of each person and his emotional state, suggesting that mPFC may make more fine-grained discriminations than personality models. It is possible therefore that the four personalities in Hassabis et al. (2014) represented four different contexts that informed participants’ expectations about the agents’ reactions.

If the pSTS is not involved in re-evaluating contextual information, then what might explain the commonly observed increase in activity to unexpected actions? Given that this region also shows greater response to attention reorienting tasks (Corbetta et al., 2008; Lee and McCarthy, 2014), the increased activity could reflect attention reorienting, or prediction error signals (Koster-Hale and Saxe, 2013). One study, however, dissociated attention reorienting from stimulus evaluations and suggested that the pSTS at the temporoparietal junction is involved in stimulus evaluation instead of reorienting attention (Han and Marois, 2014). Therefore, the increased activity could also reflect greater stimulus evaluation, given the unexpectedness of the stimulus.

Another possibility is that the pSTS represents the expected outcome (e.g., a specific action), and when the outcome violates expectations, the region re-represents the outcome, leading to increased activity. However, we also found no evidence that the pSTS could discriminate between expected outcomes (in this case, the meat dish or vegetable dish) in this study. Indeed, the target object of an agent’s reach was found to be encoded in the left IPS instead (Hamilton and Grafton, 2006). It is also possible that the pSTS’ representation of expected outcomes could be specific to the domain of motion information and not static pictures as was used here, especially since the pSTS is known to respond robustly to biological motion (Allison et al., 2000; Puce and Perrett, 2003). For example, Said et al. (2010) found successful discrimination of dynamic facial expressions in the pSTS. However, motion was not presented in this study because our aim was to investigate the expectation phase of observation with no feedback, and goal-directed motion would inevitably hint at an outcome. A study that investigates if the pSTS can discriminate between different *expected* actions can address this issue.

### Limitations

One limitation of this study is the rule-based nature of the task. That is, participants could have learned and applied the face-emotion-food combinations without reflecting on the person’s goal. The left IPL/IPS has been found to represent event-specific (i.e., specific word-picture pairings) information (Kuhl and Chun, 2014), which resembles the face picture and emotional word pairings in the current study. We did not, however, find successful classification of the two types of “No Preference” trials, which suggests that there was additional information being represented in the four-way classification than just the face-emotion combination (perhaps the more subtle face-emotion-word combination). Other studies have also found decoding of task rules in the IPS (Woolgar et al., 2011; Zhang et al., 2013). It is possible, though, that the same mechanisms underlie action observation. For example, in Vander Wyk et al. (2009, 2012), an observer who sees a person scowling at an object presumably expects the person to retrieve the other object due to some internal rule, for example, Bayesian models for cue integration (Zaki, 2013).

### Conclusion

In summary, we found no evidence that the right pSTS, a region that has been shown to be sensitive to the context in which the observed movements of others occur, discriminates contextual information. We did, however, identify a network of other brain regions commonly associated with context processing and semantic and memory retrieval that successfully discriminated contexts. These regions possibly support the formation of context-dependent expectations of an agent’s goal.

## Conflict of Interest Statement

The authors declare that the research was conducted in the absence of any commercial or financial relationships that could be construed as a potential conflict of interest.

## Footnotes

^1^http://surfer.nmr.mgh.harvard.edu/optseq

^2^http://www.fmrib.ox.ac.uk/fsl
